# Surgical Treatment of Penile Verrucous Squamous Cell Carcinoma in an HIV-Infected Patient: A Case Report

**DOI:** 10.7759/cureus.37762

**Published:** 2023-04-18

**Authors:** Yacoub A Okieh, Craig Aurtz Tombet, Anouar El Moudane, Yousef Kouiss, Ali Barki

**Affiliations:** 1 Urology, Faculty of Medicine and Pharmacy of Oujda, Oujda, MAR; 2 Urology, Mohammed VI University Hospital, Oujda, MAR

**Keywords:** infection, surgery, verrucous carcinoma, total penectomy, human immunodeficiency virus

## Abstract

Although uncommon, penile carcinoma can be a debilitating disease with various causes, and cancer is a significant contributor to morbidity and mortality in individuals infected with HIV. Verrucous carcinoma, a subtype of epidermoid carcinoma, is typically slow-growing and has a low propensity to metastasize. We present a case study of a 55-year-old HIV-positive patient with a massive squamous cell carcinoma of the penis that had been developing for over two years. To treat the condition, the patient underwent a total penectomy, perineal urethrostomy, and bilateral inguinal lymphadenectomy.

## Introduction

Penile cancer is a rare form of cancer that affects males and is less common compared to other types of urological malignancies, with a reported incidence rate of 0.1-8.3 cases per 100,000 males [1]. The annual incidence in the United States and Europe is less than 1 per 100,000 males [1]. The incidence is highest in Brazil, Uganda, and India, while the lowest rates are found among Jewish and Muslim communities where male circumcision is predominantly practiced during infancy and childhood [2]. Research has shown that circumcision in early childhood can reduce the risk of penile cancer by three to five times, likely due to its protective effect against chronic irritation [2]. Notably, circumcision is widely practiced in Morocco.

## Case presentation

**Figure 1:**
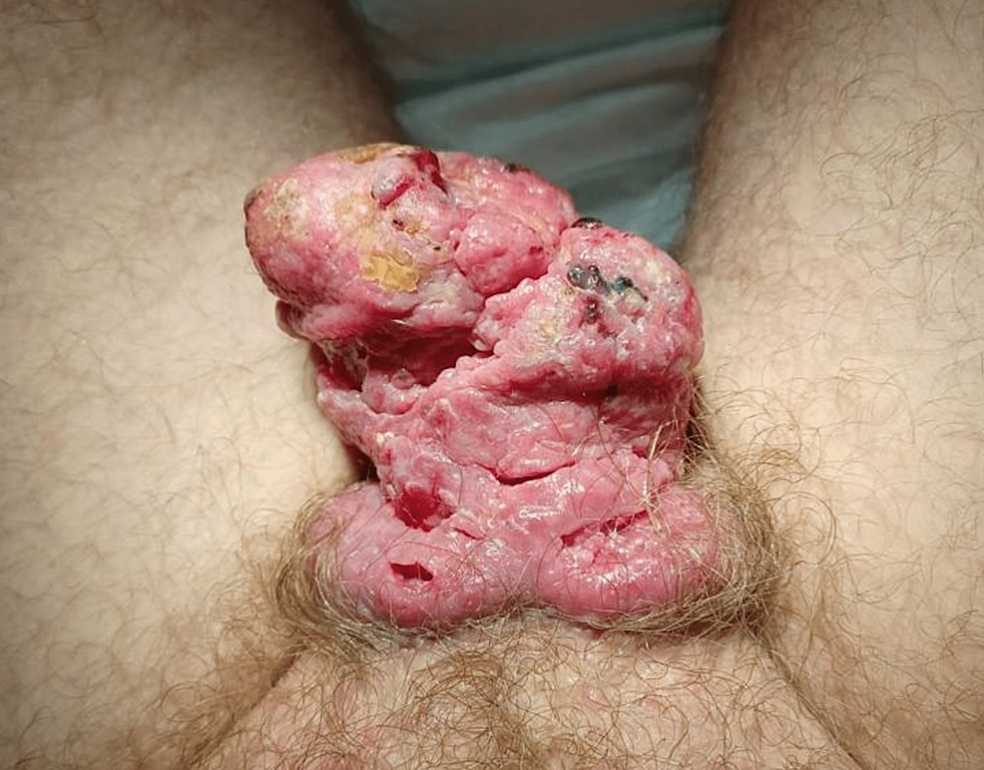
Penile mass on physical examination.

A 55-year-old uncircumcised man was admitted to the ER for acute urinary retention with a painful penile mass. Despite having had the mass for two years, he had not sought medical attention due to psychosocial factors. His medical history revealed he was HIV-positive and on antiretroviral treatment, having had several previous sexual partners. He was receiving regular medical treatment for hypertension and diabetes. Upon physical examination, it was observed that the condition of the penis had deteriorated. An ulcerative mass was present, which occupied the entire penis, and a bladder globe was noted (Figure 1).

**Figure 2:**
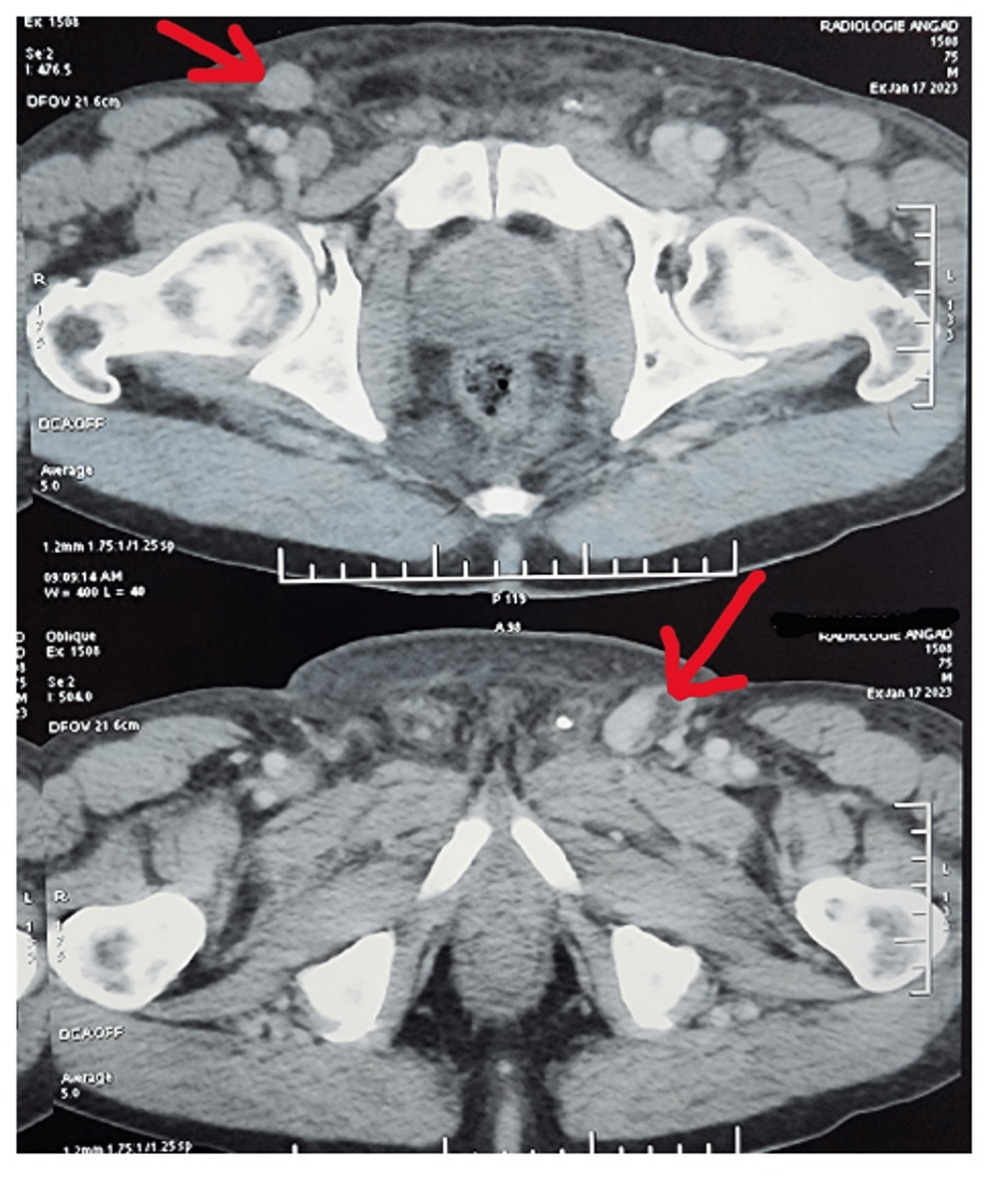
CT scan of the thorax, abdomen, and pelvis revealed bilateral inguinal lymphadenopathies.

Multiple enlarged palpable inguinal nodes were noted with variable size, the largest being 2 centimeters on the right, and consistency was hard and present on both sides. Laboratory test results were normal, and PCR testing for human papillomavirus DNA was negative. The patient underwent emergency bladder drainage with a suprapubic catheter. A CT scan of the thorax, abdomen, and pelvis was performed, revealing bilateral inguinal lymphadenopathies from 2 to 3 cm but no evidence of metastatic disease in the penile region (Figure 2).

**Figure 3:**
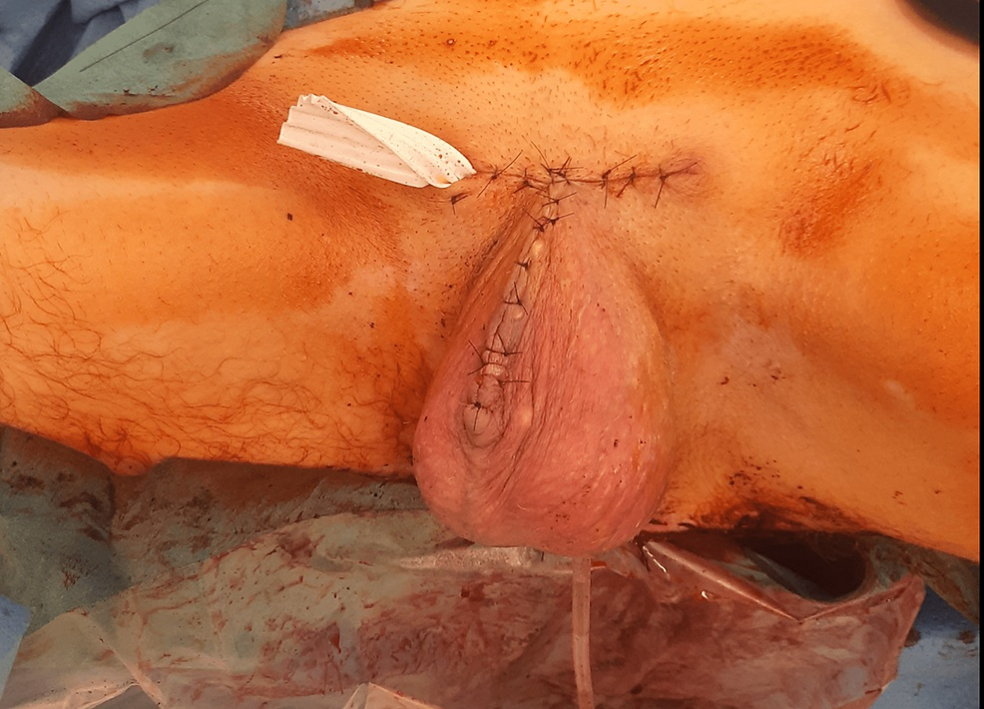
The surgical area after total penectomy and perineal urethrostomy were performed.

Under continuous spinal anesthesia, the patient underwent total penectomy with a perineal urethrostomy and bilateral inguinal lymphadenectomy, along with the removal of the suprapubic catheter (Figure 3).

**Figure 4:**
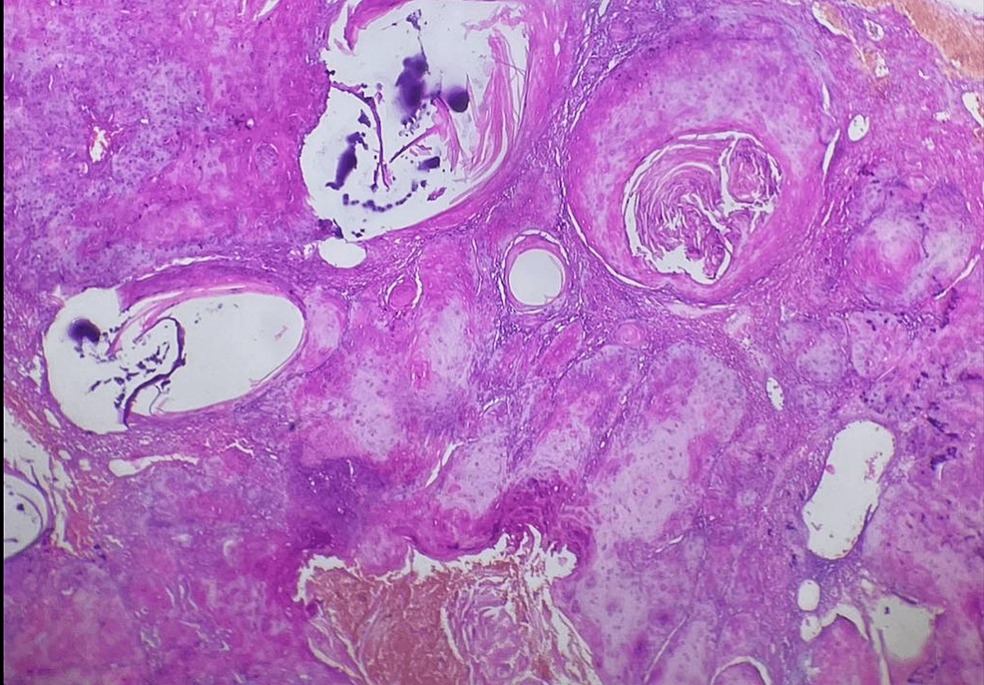
H&E-stained sections of the defects revealed squamous cell carcinoma with signs of keratinization.

Histological analysis confirmed that the mass was a well-differentiated verrucous squamous cell carcinoma of the penis that had involved nearby skin, while the surgical margin was negative (Figure 4).

The inguinal lymph nodes showed inflammatory lymphoid hyperplasia without metastatic disease, and there was no evidence of distant metastasis during the two-year follow-up.

## Discussion

Penile cancer is a rare cancer affecting less than 1% of men [3]. It occurs more frequently in individuals with phimosis as opposed to those with a redundant foreskin. Risk factors for the disease include being uncircumcised, having balanoposthitis or balanitis xerotica obliterans (BXO), exposure to ultraviolet phototherapy, engaging in sexual promiscuity, having early sexual intercourse, having a history of condyloma, smoking tobacco, and having sexual intercourse with a human papillomavirus-infected partner [4]. The patient in our case report was uncircumcised. Furthermore, studies have linked the pathogenesis of penile cancer to human papillomavirus [5]. Verrucous squamous cell carcinoma was first described by Ackerman in 1948, who identified it as occurring in the oral cavity. It represents 20% of verruciform lesions and 3-8% of penile cancers. This uncommon subtype presents as an exophytic, papillomatous, low-grade, well-differentiated tumor. Nevertheless, diagnosis may pose a challenge since a diagnostic biopsy could produce inaccurate results [5]. The most frequent cancers that affect individuals with HIV infection are those categorized as AIDS-related, including Kaposi's sarcoma, non-Hodgkin's lymphoma, and invasive cervical cancer [6]. Nevertheless, other forms of cancer, such as Hodgkin's disease, anal cancer, lung cancer, and testicular germ cell tumors, seem to occur more commonly in HIV-infected individuals than in the general population [6]. The principal approach to treating verrucous carcinoma typically involves surgical intervention, which may entail a total or partial penectomy, along with or without supplementary chemotherapy. Alternative treatment options include Mohs surgery, CO2 laser surgery, and liquid nitrogen cryosurgery for small lesions, either alone or in combination with topical fluorouracil (5-FU) . Additionally, systemic or intralesional treatment with interferon-α in combination with a surgical shave may be used. A surgical strategy is usually favored due to its ability to produce excellent treatment outcomes, facilitate comprehensive histological sampling, and permit investigation for focal squamous cell carcinoma [7].

## Conclusions

Penile cancer is a highly uncommon type of male genitourinary cancer that typically originates in the epithelium of the inner preputial wall or glans. Primary etiological factors include phimosis, poor penile hygiene, sexually transmitted infections, and smoking. Squamous cell carcinoma accounts for nearly 95% of penile cancers, with onset usually occurring in the sixth decade of life. The primary mode of treatment involves surgical resection, with partial or total penectomy being the established surgical approach. The most critical prognostic factor remains the presence of inguinal lymph node metastases, which are strongly associated with distant metastases.
